# Complex Ecological Dynamics and Eradicability of the Vector Borne Macroparasitic Disease, Lymphatic Filariasis

**DOI:** 10.1371/journal.pone.0002874

**Published:** 2008-08-06

**Authors:** Manoj Gambhir, Edwin Michael

**Affiliations:** Department of Infectious Disease Epidemiology, School of Medicine, Imperial College London, London, United Kingdom; Duke University, United States of America

## Abstract

**Background:**

The current global efforts to control the morbidity and mortality caused by infectious diseases affecting developing countries—such as HIV/AIDS, polio, tuberculosis, malaria and the Neglected Tropical Diseases (NTDs)—have led to an increasing focus on the biological controllability or eradicability of disease transmission by management action. Here, we use an age-structured dynamical model of lymphatic filariasis transmission to show how a quantitative understanding of the dynamic processes underlying infection persistence and extinction is key to evaluating the eradicability of this macroparasitic disease.

**Methodology/Principal Findings:**

We investigated the persistence and extinction dynamics of lymphatic filariasis by undertaking a numerical equilibrium analysis of a deterministic model of parasite transmission, based on varying values of the initial L3 larval density in the system. The results highlighted the likely occurrence of complex dynamics in parasite transmission with three major outcomes for the eradicability of filariasis. First, both vector biting and worm breakpoint thresholds are shown to be complex dynamic entities with values dependent on the nature and magnitude of vector-and host specific density-dependent processes and the degree of host infection aggregation prevailing in endemic communities. Second, these thresholds as well as the potential size of the attractor domains and hence system resilience are strongly dependent on peculiarities of infection dynamics in different vector species. Finally, the existence of multiple stable states indicates the presence of hysteresis nonlinearity in the filariasis system dynamics in which infection thresholds for infection invasion are lower but occur at higher biting rates than do the corresponding thresholds for parasite elimination.

**Conclusions/Significance:**

The variable dynamic nature of thresholds and parasite system resilience reflecting both initial conditions and vector species-infection specificities, and the existence of hysteresis loop phenomenon, suggests that eradication of filariasis may require taking a more flexible and locally relevant approach to designing elimination programmes compared to the current command and control approach advocated by the global programme.

## Introduction

The current global efforts to control the morbidity and mortality caused by infectious diseases affecting developing countries—such as HIV/AIDS, polio, tuberculosis, malaria and the Neglected Tropical Diseases (NTDs) [1]—have, apart from giving rise to new thinking regarding how best to organize, manage and finance disease control at large population and inter-population scales, led to increasing focus on the biological controllability or eradicability of disease transmission by management action [2], [3]. A key research topic in this connection for diseases such as lymphatic filariasis, where the intervention objective is eradication of transmission, has been the garnering of better knowledge of the dynamics of parasite transmission and extinction, including determining if infection thresholds below which the disease is driven to extinction exist at levels which are operationally significant [4], [5]. This has now become critical for this disease in particular, given the urgent need for management to be able to specify reliable endpoint targets signifying achievement of the goal of parasite eradication following the institution of currently recommended interventions [6]–[8].

It has long been known from the analysis of mathematical models of parasite transmission that the occurrence of dynamic interactions between structural components and non-linearities, such as density dependences, in parasite systems dynamics can introduce complex phenomena, including threshold infection levels in the host population or vector biting rates (in host-vector-parasite systems), the crossing of which can cause the parasite system to switch to alternate stable states wherein the parasite population either becomes extinct or stabilizes to an endemic level [9]. Moreover, this work has shown how, if several density dependences exist in a system, these could additionally interact significantly to influence threshold behaviours [10], and hence stability, resilience and change from one state to another, in parasite systems. These considerations indicate that gaining a better understanding of the nature and processes which underlie parasite transmission dynamics will—by revealing alternate regime states and their respective sizes, system points of change, and nature of the regime shifts— be not only fundamental to the detection and quantification of elimination thresholds but also as a consequence the effective management of disease eradication.

Here, we extend recent work [11], [12] in developing and analyzing an age-structured dynamical model of lymphatic filariasis transmission in order firstly, to clarify whether complex system dynamics may govern parasite transmission, and secondly, to uncover the processes that could underlie such dynamics and hence contribute to the persistence, resilience and extinction of this macroparasitic infection. A practical objective was to derive and characterize the two eradication thresholds that are likely to underlie the system dynamics of this vector-borne infection, in order to provide and assess their values as endpoint targets for the global filariasis elimination programme currently being or planned to be implemented in endemic countries [10].

Two distinctive density dependences are included in the model in accordance with available data and knowledge regarding infection processes governing parasite transmission dynamics: one which governs the uptake of microfilariae (Mf) and the development of L3-stage (infective) larvae by mosquito vectors when a bloodmeal is taken from a human host [13]–[15]; and a second concerning the mating probability of worms inside the human hosts [16]. We regard that both these density dependences are likely to independently and simultaneously act to affect the transmission of filariasis, via the introduction of vector biting transmission thresholds and breakpoint worm levels in the human population [10]. The effects of another density-dependent mechanism, human acquired immunity, and parasite distribution among hosts on the magnitude of obtained thresholds are also explored. A key advance on previous studies [11], [12] is that we have also investigated here for the first time the consequences of these processes on filarial extinction dynamics and thresholds in the two major parasite-vector species combinations implicated in the global transmission of this parasite: viz. one in which the vector intermediate host are culicine mosquitoes and the other in which they are anopheline. The import of our results for the global lymphatic filariasis eradication programme are discussed in relation to the dynamical nature of extinction thresholds and system stability and resilience, the influence of vector species, and the nature of control management required given these system dynamics for achieving the successful eradication of this parasitic disease.

## Materials and Methods

### Outline of the model

Lymphatic filariasis is a parasitic disease caused by filarial nematode worms affecting some 120 million humans in tropical and subtropical areas of Asia, Africa, the Western Pacific and some parts of the Americas [17]. The filarial parasites have biphasic life cycles involving the definitive mammalian host and various genera of transmitting mosquito vectors. Adult worms inhabit the human host lymphatics, where they sexually reproduce and give rise to larval transmission stages, called microfilariae. These enter the host peripheral blood circulation where they are available to be ingested by mosquito vectors during a blood meal. The microfilariae then undergoes further larval development in the vector hosts to become infective L3-stage larvae, and the parasite life cycle is completed when these infect human hosts at the next blood meal taken by the vector. The deterministic dynamical model for lymphatic filariasis transmission thus primarily consists of a series of coupled differential and partial differential equations for three state variables describing the changes in numbers over time of the above key parasite life stages—worm burden (*W*), Mf count (*M*), and stage L3 larvae (*L*)—and one state variable describing the acquisition and loss of immunity to parasites in human hosts (*I*) [11], [12], [18]. Structurally, these equations are divided into those which describe changes in parasitic infection in the human definitive hosts (*W*, *M*, *I*), and the L3 larval equation (*L*), which is used to calculate the change in larval density in the mosquito intermediate hosts. The equations pertaining to changes in parasite stages in the human host population describe change as a function of host age as well as time, since model inputs—such as exposure to mosquito biting—vary over age. By contrast, because the processes governing the rate of change of the average L3 larval density within the mosquito population operate on much shorter timescales than those in the human host population, the model assumes that the mosquito larval density comes to equilibrium as soon as the corresponding human host parasite and immunity levels are computed. The coupled partial differential and differential equations comprising the dynamical model are thus:
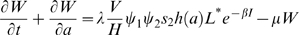
(1)

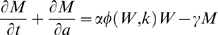
(2)

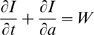
(3)

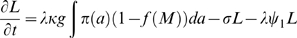
(4)The above equations are similar to those previously published for the lymphatic filarial transmission model described by Norman *et al* [12], although here a mating probability (*φ*(*W*,*k*) = 1−(1+*W*/2*k*)^−(1+*k*)^, [16]) has been incorporated into the mean Mf count (*M*) equation to account for the production of Mf as a function of density-dependent worm mating within the human hosts. This probability function is dependent upon the worm burden *W* and the shape parameter *k* of the negative binomial distribution describing parasite dispersion among hosts, with the form selected to account for the dioecious and polygamous nature of *Wuchereria bancrofti* worms [8], [16]. The dependence of the shape parameter of the negative binomial distribution on the average worm burden is considered to be identical to that used to describe the relationship between Mf prevalence and intensity, i.e. it is a linear function of worm burden with gradient *k*_1_ and with zero intercept *k*_0_ (see below and Table 1).

**Table 1:** Description and values of the parameters of the model.

Parameter symbol	Definition	Value	Source
λ	Number of bites per mosquito	10 per month	[45], [46]
*V/H*	Ratio of number of vectors to hosts	Systematically varied over model runs	n/a
*ψ*_1_	Proportion of L3 leaving mosquito per bite	0.414	[47]
*ψ*_2_	Proportion of L3 leaving mosquito that enter host	0.32	[48]
*s*_2_	Proportion of L3 entering host that develop into adult worms	0.2	Ash (1974) WHO/FIL/74.121, *World Health Organisation report*
*β*	Strength of acquired immunity	0.112	[12]
*μ*	Death rate of adult worms	0.0104 per month	[49]–[52]
*α*	Production rate of Mf per worm	0.2 per month	[47]
*γ*	Death rate of Mf	0.1 per month	[47], [50]
*g*	Proportion of mosquitoes which pick up infection when biting an infected host	0.37	[53]
*σ*	Death rate of mosquitoes	5 per month	[48]
*k(M)*	Aggregation parameter from negative binomial distribution	*k*_0_+*k*_1_ *M* (0.0029+0.0236*M*)	[54], [55]
*h(a)*	Parameter to adjust rate at which individuals of age *a* are bitten: linear rise from 0 at age zero to 1 at 10 years	Varying*	[12]
*L* ^*^	Equilibrium value of the larval density (see Equation 5)	Varying*	n/a
*φ*(*W*,*k*)	Mating probability as a function of worm burden *W* and aggregation parameter *k*	Varying*	[16]
*π*(*a*)	Probability that an individual is of age *a*	Varying*	[12]
*f(M)*	Variable component of the population-averaged Mf uptake and L3-larval development function; this is a function of the average Mf level *M* (see Equations 8 and 10 below for specific functional forms)	Varying*	n/a

* The values referred to here as ‘varying’ are either functions of quantities changing over simulation runs or multi-valued parameters, described in the Definition field.

Since the rates associated with the vectors are considerably faster than the time taken in the development of worm and Mf burdens, the L3 density is assumed to instantaneously equilibrate. By setting the right hand side of Equation 4 to zero and rearranging, the following expression is obtained for the instantaneous value of the L3 larval density, *L* ^*^:
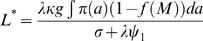
(5)Here, the parameter *κ* denotes the saturation value of the vector mf uptake function, and is explained in detail below. Table 1 provides a description of the equation parameters along with the values used in the simulations.

### Uptake of Mf and development of L3 larvae within vectors

When mosquitoes, acting as intermediate hosts, alight upon a human host and take a blood meal, they ingest a number of Mf from the definitive host, some or all of which may develop within the mosquito into L3 larvae. There are three functional forms—facilitation, limitation or proportionality—that have been found to describe the relationship between the number of Mf ingested and the number that develop within the mosquito into an appropriate stage for egestion when the vectors next bite humans [14], [15].

**Figure 1:**
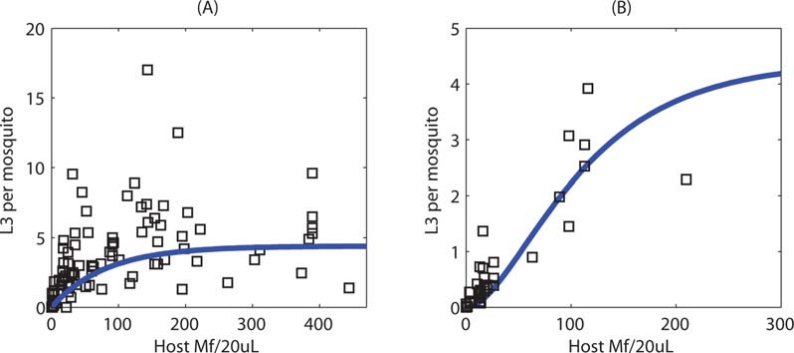
Functional forms relating microfilaria (Mf) uptake (from 20 µL host blood) and L3 development per mosquito in each vector genus. The squares denote observed data for each vector respectively (sources outlined in the text). The curve fitted to the (A) *Culex* data is a limiting function of Mf (equation 6 in the text) whereas the *Anopheles* curve in (B) describes a development response that begins with a facilitation phase which then approaches an upper limit at higher Mf uptakes (equation 9 in the text).

The impact on the statics and dynamics of the host-vector-filarial parasite system in this study was analysed for the two major vector genera implicated in the transmission of lymphatic filariasis, viz. culicine and anopheline mosquitoes. These two genera have been shown to produce different Mf to L3 uptake and developmental behaviours. For *Culex* mosquitoes, a limitation response occurs which results in a saturation in the production of L3 larvae as Mf loads in blood meals increase, while for *Anopheles* mosquitoes, a positive density-dependent Allee-type effect is thought to exist that, beyond a critical Mf threshold at low burdens, acts to “facilitate” L3 production but below which acts to hamper the development of this larval stage [14]. *Culex* uptake data can thus be fitted to the function [12]:
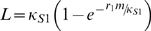
(6)where *m* is the Mf density in the human host (20 µL blood), *κ_S_*_1_ is the maximum limiting value of L3 numbers developing in the mosquito, and *r*_1_ controls the rate at which the L3 development rises with Mf ingested. The fit of this function to data collected from all available published studies [13], [14] is shown in Figure 1A. Note that while this is the appropriate functional form for the uptake of Mf and the development of L3 mosquitoes in a single mosquito from one blood meal, it is necessary to account for the effect of the distribution of Mf among hosts in order to quantify the average L3 output in a community. In order to do this, the average L3 level is calculated in the mosquito populations when they take blood meals from a human host community in whom the mean Mf level is *M* and is assumed to be negative binomially distributed with shape parameter *k*. Appendix S1 outlines how, by using the moment-generating function of the negative binomial distribution, and the fact that its probabilities sum to 1, this population-averaged uptake function (*L_pop_*) can be derived as:
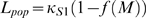
(7)where:
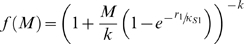
(8)This function is very similar to the individual uptake curve, but there is now a dependence on the shape parameter *k*, which determines the level of parasite aggregation in the human hosts.

For anopheline mosquitoes, a closely-related function to that used to model the *Culex* uptake response, but describing behaviour consistent with facilitation in the part of their Mf-L3 uptake curves corresponding to low Mf densities, can be given by the equation:
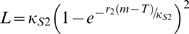
(9)As for the *Culex* function, *κ_S_*_2_ is the maximum limiting value of L3 numbers developing in the mosquito, and *r*_2_ controls the rate at which L3 development rises with Mf ingested. This function has two features that distinguish it from the *Culex* function above. The first is the fact that it is raised to the power of two, an operation that introduces a concavity into the shape of the function prior to its limitation-associated saturation at very high Mf loads. The second is the offset parameter (*T*) appearing in the exponent of the exponential function, which ensures that the uptake value rises smoothly from its zero point only when the Mf density in a blood meal is greater than a threshold density given by the value described by *T*. Biologically, this functional behaviour may be associated with the action of the anopheline cibarial armature or teeth, which prevents Mf from passing undamaged into the mosquito gut at intakes lower than the threshold *T* [15] but which allows the undamaged passage of the majority of ingested Mf at higher ingestion loads owing to the protection afforded due to the entanglement of the first-passing Mf about the cibarial teeth . The best-fit of the model to published data is shown in Figure 1B. Again, this function is averaged over the host population and the following population-averaged function is obtained (see Appendix S1 for derivation):
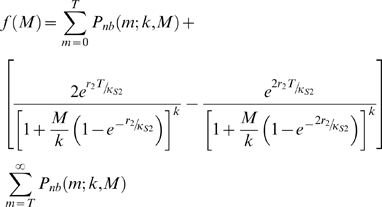
(10)Where *P_nb_*(*m*;*k*,*M*) is the negative binomial probability mass function, with mean *M* and aggregation parameter *k*. Table 2 provides the best-fit parameter values for both the *Culex* and *Anopheles* functions given in Equations 6 and 9.

**Table 2:** The parameter values used in the model for the vector uptake and development functions. These values were obtained through nonlinear least squares fits from the data collated by Snow and Michael [13].

Parameter	Value	Standard Error
*κ_S_*_1_	4.406	0.362
*r*_1_	0.019	0.058
*κ_S_*_2_	4.395	0.332
*r*_2_	0.055	0.004
*T*	0	n/a

### Investigating system dynamics: multiple stable states, transitions and extinction dynamics

We investigated the persistence and extinction dynamics of the parasite system using numerical simulations of the model based on varying initial values of L^*^. The following steps were used to evaluate the equilibria of the model: 1) initialise the average L3 larval level and integrate the state equations over age, 2) calculate the new L3 larval level implied by the corresponding age-dependent host distribution of Mf using the L* formula for the instantaneous equilibrium value of L3 larvae (Eq. 5), 3) calculate the corresponding average Mf intensity in the population using the age distribution of Mf and the population age-distribution, 4) re-insert the new L3 level into the equations and integrate over age for host infection, and 5) repeat steps (1) to (3) until the difference between the L3 larval level L* at the most recent step and the previous one is less than a tolerance value of 10^−8^. This procedure is, therefore, a numerical analysis of the stability of the solutions of the model equations, i.e. when the rate of change of each state variable becomes zero, where initial values of L3 are supplied. Each age-dependent integration of the model was computed using the Euler integration method in Matlab®.

Worm breakpoints in the human host are investigated in this approach as follows. The vector biting rate is progressively increased from zero and, for each biting rate value, the model is initialised with values of the L3 larval level beginning at zero and increasing in small increments. Low values of the initial L3 larval level will result in the model converging on the zero-parasite equilibrium, while high values of the initial level will result in the endemic equilibrium (as long as the vector biting rate exceeds the TBR). In between these low and high values, there exists an L3 value below which the system will converge to zero, and above which it will converge to the endemic level, therefore giving rise to the breakpoint L3 larval level. As each L3 level is uniquely identified with a corresponding population Mf load, worm breakpoints are thereby also readily uncovered using this approach. In all the analyses that follow, these Mf loads are converted into population Mf prevalence levels using the expression *P*(*M*,*k*) = 1−(1+*M*/*k*)^−*k*^, where *P* is the prevalence, *M* is the Mf density and *k* is the aggregation parameter of the negative binomial distribution given that Mf prevalence rather than intensity is the normal infection variable measured in the field. Thus, each model run is started with a specific value of the L3 larvae and, if this leads to the zero equilibrium, either the Threshold Biting Rate (TBR)—the vector biting rate below which no disease transmission occurs—has not been reached or the initial L3 level lies beneath the parasite breakpoint (in terms of Mf prevalence). If a non-zero equilibrium is achieved, then the initial L3 larval density lies above the breakpoint and an endemic state has been discovered.

### Basins of attraction and system resilience

We incrementally perturbed the two model systems with increasing initial L3 values around representative worm breakpoint values to investigate the relative magnitudes of change required before the Mf age-prevalences for each system stabilised at either the zero- and endemic equilibrium attractors. Each successive L3 value used for the perturbations was obtained iteratively from the numerical stability analysis procedure outlined in the previous section. These simulations thus also afforded simple assessments of the sizes of the respective basins of attraction to either the zero- or endemic equilibrium points, and therefore a first examination of the resilience, i.e. the magnitude of perturbations a system can withstand before it changes stable states , [19]–[21], of each system to either stable state. Furthermore, given that the successive L3 values used in the perturbations were obtained from model convergences to stable states, the solutions from each perturbation run until stable Mf age-prevalences are reached also afforded a preliminary evaluation of the rates at which the two model systems change from the vicinity of each steady state.

### System sensitivity

The sensitivity of the estimated equilibrium Mf prevalence-vector biting rates to three important model parameters that are likely to depict significant variation between communities was explored by quantifying the changes produced as a result of varying the values of these key parameters. The three parameters investigated were the degree of community infection aggregation as described by the location parameter of the negative binomial distribution *k*, whose linear component was varied by 50%; the strength of acquired immunity to worms, β, which was varied by 10%; and per capita worm fecundity, α, whose value was doubled. In each case, the endemic and zero equilibria, breakpoints, and TBR were found.

### Hysteresis

We examined hysteresis effects in the models by determining patterns of worm breakpoints and system shifts to alternate stable zero- or endemic infection states as initial L3 values are varied [21].

## Results

### Equilibria, bifurcations and stable states

**Figure 2:**
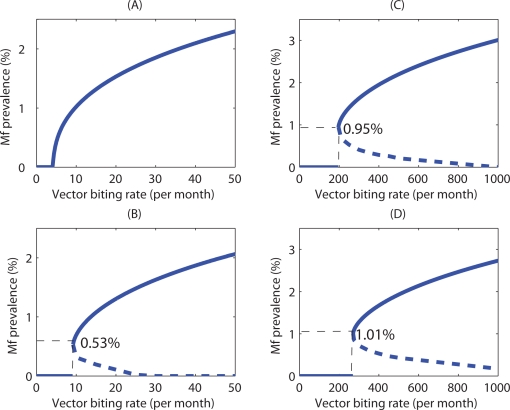
The effect of varying the vector biting rate on the equilibrium Mf prevalence among human hosts when the vector intermediate host was culicine and when the worm mating probability was (A) and was not (B) included in the model, and when the intermediate host was anopheline with (C) no mating and with (D) mating probabilities included. Inclusion of the mating probability introduced a set of breakpoints (dotted line) in the case of *Culex*, and it raised and increased the range of the breakpoints in the case of *Anopheles*. The labelled Mf values on the y-axes correspond to the maximum breakpoints, whereas additionally in each graph the solid curve (A) and vertical dashed drop lines (B–D) crossing the x-axes denote the threshold biting rates (TBRs) estimated in each scenario corresponding to A) 4, B) 9, C) 197, D) 271 vector bites per month.

The results from the numerical equilibrium analyses described in Materials and Methods for both the culicine and anopheline models are shown in Fig. 2. The graphs depict the steady states of host Mf infection prevalence as well as the nature of their transitions that appear along the vector biting rate gradient with and without the inclusion of worm mating probabilities in the respective models. Thus, in the culicine case, when worm mating probabilities were not included and where the L3 uptake and development function shows only limiting behaviour, the results show that the system gives rise to just one threshold - a vector biting rate below which the only stable equilibrium was at the zero parasite level (Fig. 2A). Above this transmission threshold, a transcritical system bifurcation appears to occur leading to the existence of endemic stable infection levels that increase in magnitude smoothly and reversibly as biting rates increase. Thus, no worm breakpoints and hence existence of alternate infection states are possible, i.e. only positive endemic states occur above the TBR, the system losing its stable endemic infection state smoothly to converge into a zero-infection state as the vector biting rate is reduced below the TBR (Fig. 2A).The effects of introducing the worm mating function into the culicine model is shown in Fig. 2B. In contrast to the situation when the function was excluded, it is clear that this can bring about a discontinuous jump in the Mf prevalence at the TBR giving rise to the appearance of three equilibria, two stable ones separated by an unstable one, mimicking thus the occurrence of a subcritical bifurcation in the system dynamics. The unstable equilibrium points of the model comprise a set of positive-valued worm breakpoints—the largest of which was found at the TBR— producing an unstable dynamic boundary or separatrix with increasing biting rates across which the system converged to either a zero or endemic stable equilibrium state. Thus, at vector biting rates above the TBR, bistable infection states, one endemic and the other zero-infection, can exist depending upon whether initial L* load values can give rise to infection levels above or below the unstable breakpoint values (Fig. 2B). By contrast, in the anopheline-mediated system in which the L3 development function was of a facilitation form, a subcritical bifurcation was observed in which a discontinuous jump in the Mf prevalence at the TBR occurs regardless of whether the worm mating function is included or not (Fig. 2C,D). The values of the vector biting rate at which the non-zero worm breakpoints exist were also far greater in this case than for the culicine system (Fig. 2B vs C,D). In addition, both the TBR and the maximum worm breakpoint values were found to be raised by the inclusion of the mating probability function.

### Perturbations, sizes of basins of attractions and system resilience

**Figure 3:**
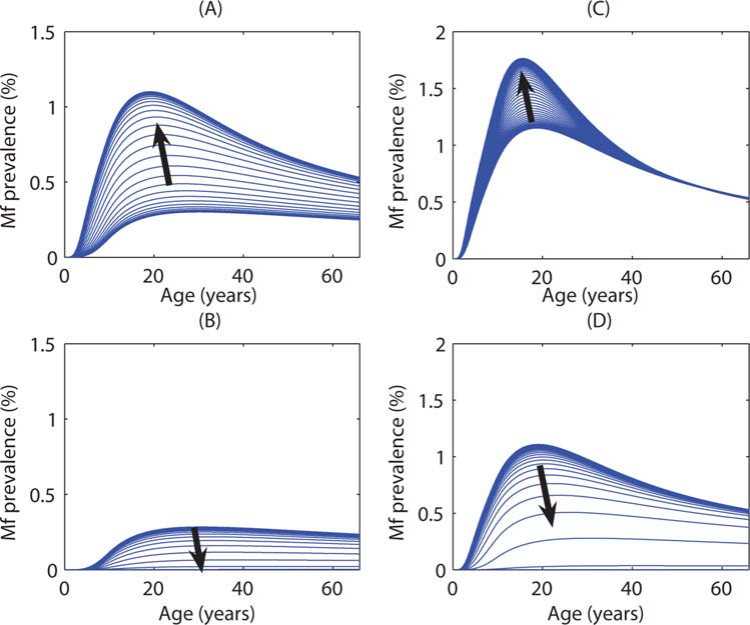
The direction of change of the Mf prevalence age-profile among human hosts when the intermediate host was culicine and the initial Mf prevalence was (A) above and (B) below the breakpoint value (0.21%) with a vector biting rate of 11 per month; and when the intermediate host was anopheline with initial Mf prevalence (C) above and (D) below the breakpoint value (0.82%) with a vector biting rate of 280 per month. Each age-dependent curve represents a perturbation around the initial unstable equilibrium curve with the black arrows indicating the direction in which these curves are likely to travel on the way to the stable equilibrium.

The results of perturbing the *Culex* and *Anopheles* models by varying initial L3 values from the vicinity of the respective unstable equilibria are illustrated in Figure 3. Each age-dependent Mf prevalence curve in these graphs represents the solution of the model equations for a given initial L3 seed value set to a value either above or beneath the breakpoint level for a vector biting rate above the estimated TBR. The results depict that an increasing perturbation below the breakpoint curve leads to an eventual age-Mf prevalence of zero across all ages, whereas a similar perturbation above the breakpoint leads to stabilisation at the equilibrium endemic level (Fig. 3). The arrows in each figure indicate the directions in which perturbations from the breakpoint will tend to change each system. An indication of the rates of system change for each model from the vicinity of the respective steady state age-Mf prevalence levels is also given by the clustering of the age-curves shown in Figure 3. Where the curves cluster—i.e. where the curves are very close together—the prevalence profile can be considered to be changing slowly, compared with more rapid changes occurring where the curves are more sparsely distributed. The results show that slow regions occurred close to the steady states for both vector-host-parasite systems (Fig. 3).

The sizes of the regions between the unstable and either stable equilibrium state are depicted in Figure 3 for each model, and clearly provide a qualitative measure of the extents of the respective basins of attraction to either stable state. As they also in turn afford a measure of the maximum amount each system can be changed before losing its ability to recover to either stable state, these results also graphically offer an insight into the likely resilience of the two parasite systems to each of the stable state. For example, the results show that in culicine filariasis, the basin of attraction to the endemic equilibrium state is much larger in size compared to the case in anopheline filariasis, while the opposite is true in the case of the basins of attraction to the zero infection state (Fig. 3A,B versus C,D). The larger basin of attraction to the endemic state but the smaller basin of attraction of attraction to the zero state thus suggests that culicine filariasis may be more resilient to perturbations of the system away from the endemic attractor compared to anopheline filariasis, i.e. the former may be more resilient to extinction compared to the latter.

**Figure 4:**
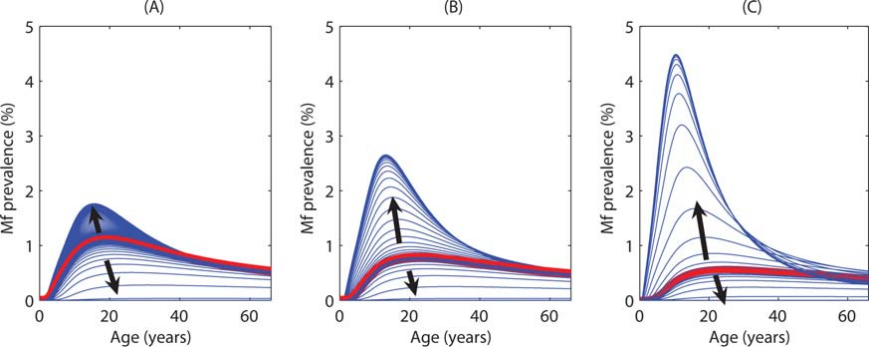
The direction of change of the Mf prevalence age-profile among human hosts at different anopheline vector biting rates. Results are shown for vector biting rates of (A) 280 (just above the TBR of 271), (B) 350, and (C) 600. In each case, the blue curves represent initial perturbations around the Mf age-profile corresponding to the breakpoint Mf prevalence (red curve), and the black arrows indicate the direction in which the curves are likely to travel on the way to attaining the zero or endemic stable equilibrium.

Figure 4 portrays the effects of perturbing the anopheline system near the vicinity of the unstable worm breakpoint values (by varying initial L3 values) on the corresponding Mf age-profiles for three increasing values of the vector biting rate. For each value of the biting rate, the system was initialised as before, above and below the breakpoint Mf prevalence level (given by the red curves in Figure 4), and the direction in which the profiles move was then tracked (indicated by the arrows in the figure). The results show that as the biting rate increases, the relative sizes of the basins of attraction for the zero-parasite and endemic equilibrium can change considerably. In particular, the range of Mf prevalence values that led to the zero equilibrium diminished while those that led to the endemic equilibrium (upper curve) grew, indicating that system resilience to perturbations even for anopheline filariasis will be greater at higher vector biting rates.

### System sensitivity

**Figure 5:**
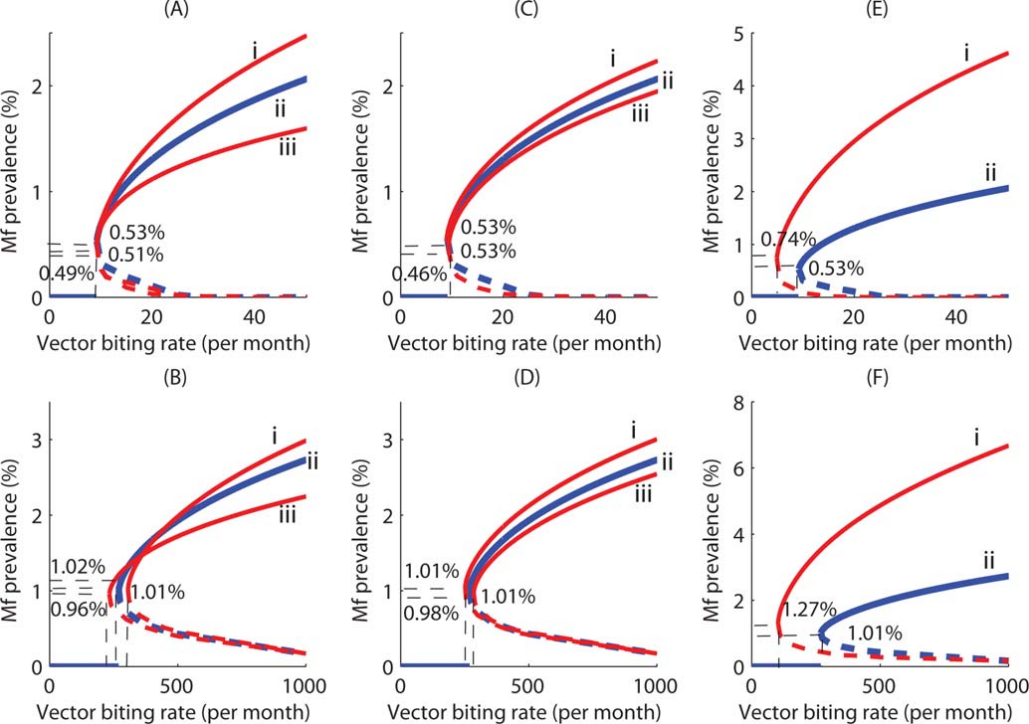
Sensitivity of the endemic Mf prevalence, breakpoint values, and TBRs to changes in the shape parameter of the negative binomial distribution, (*k*) describing the distribution of Mf among human hosts ((A) and (B)), the strength of the immune response to infection, *β* ((C) and (D)), and per capita worm fecundity, *α* ((E) and (F)). The *k* values were varied by maintaining the linear dependence upon Mf values (see text) but increasing/decreasing the linear component by 50% so that this value was given by i) 0.0354, ii) 0.0236, and iii) 0.0118. When the intermediate host was (A) culicine, the maximum value of the breakpoint changed with *k*, but the TBR (units are bites per month) did not change (TBR = 9). For anopheline intermediate hosts (B), both the maximum breakpoint and the TBR decreased with decreasing *k* (TBR = i) 306, ii) 271, iii) 233)). The parameter *β* can be thought of as an index governing the strength of the density dependent establishment of parasites in the human hosts. The *β* values were varied by 10% so that the values used here were: i) 0.1, ii) 0.112, and iii) 0.122. When the intermediate host was (C) culicine, the maximum value of the breakpoint changed with *β*, but the TBR did not change from its value of 9. For anopheline intermediate hosts (D), the maximum breakpoint decreased and the TBR increased with increasing *β* (decreasing density dependence) (TBR = i) 252, ii) 271, iii) 285)). Graphs (E) and (F) depict the steady state Mf prevalence values found for values of per capita worm fecundity, *α*, of either i) 0.4 or ii) 0.2. When the intermediate host was either (E) culicine or (F) anopheline, the maximum value of the breakpoint rose and the TBR was lowered with increasing *α* (TBR (E) *Culex*: i) 5, ii) 9; (F) *Anopheles*: i) 104, ii) 271)).

Figure 5 shows the equilibrium solutions for the Mf prevalence breakpoints and TBRs obtained for the culicine and anopheline models respectively as functions of three key variables which are likely to vary between endemic communities, viz. the degree of infection aggregation among hosts as encapsulated by the value of *k* of the negative binomial distribution (Fig. 5A,B), the strength of acquired immunity to worms, β (Fig. 5C,D), and the per capita worm fecundity, α (Fig. 5E,F). The results indicate that not only may these thresholds vary as a result of differences in the initial values or conditions of these key community infection variables but also that the effects are dependent upon the host-vector-parasite combination in filariasis transmission. Thus, while Mf prevalence breakpoint values decreased as the infection aggregation increased (*k* decreasing) in the case of both the culicine and anopheline models, an effect on TBR (increasing as infection aggregation becomes less overdispersed (increasing *k*)) occurred only in the case of the anopheline model (Fig. 5A,B). Similarly, the primary effect of increasing acquired immunity was also to depress the Mf prevalence breakpoints in the case of both vector species, although again a secondary impact on TBR (an increase with increasing immunity) was observed more markedly in the case of anophelines and only at higher levels in culicines (Fig. 5C,D). The impacts of varying per capita worm fecundity values, a key uncertainty in the models, on both thresholds are shown in Fig.5E,F. For both models, the effect of increasing worm fecundity was to increase the maximum worm breakpoints and decrease the corresponding TBRs.

### System hysteresis

**Figure 6:**
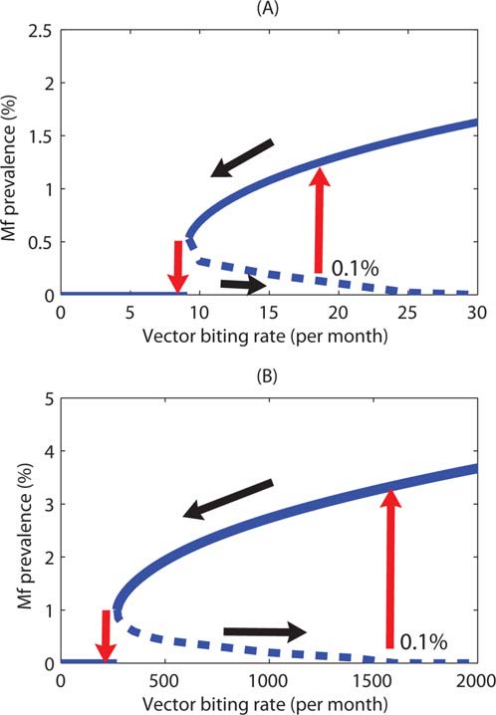
Hysteresis loops in the Mf prevalence/vector biting rate plane for (A) culicine and (B) anopheline intermediate hosts, showing the two asymmetrical ways by which a shift between alternative Mf stable states can occur with varying vector biting rates. If the parasite system is on the lower zero state but at high vector biting rates and thus close to the worm breakpoint bifurcation boundary, a slight incremental change in Mf levels may bring it beyond the birfurcation (say at 0.1% Mf prevalence) and induce a drastic shift of the system to its endemic equilibrium (rightmost red arrow). If one attempts to restore the parasite-free equilibrium state by reducing the vector biting rate (black leftward arrow), the system shows hysteresis. A backward shift to the parasite-free equilibrium (leftmost red arrow) will occur only if the vector biting rate is reduced far enough to reach the TBR bifurcation point. The hysteresis loop is wider in extent for the anopheline model compared to culicine-transmitted filariasis.

The existence of multistable states separated by an unstable boundary when the TBR has been exceeded introduces the possibility of the occurrence of bistability or hysteresis in the transmission dynamics of lymphatic filariasis [9]. Figure 6A illustrates this phenomenon in terms of the equilibrium Mf prevalence versus vector biting rate relationship for the model in which the vector is culicine. The bottom black arrow shows the path taken by the equilibrium Mf prevalence when the biting rate is increased to the point on the far right (the 0.1% Mf breakpoint). Until this point is reached, the Mf steady state remains at zero. There is a jump (a subcritical bifurcation) at this point to the endemic equilibrium stage (upward pointing red arrow), and the steady state remains on this stable branch as the biting rate is increased further. Decreasing the biting rate to a point at which the endemic state appeared, however, will not return the system to the zero Mf prevalence state. For this to happen, the biting rate needs to be reduced further backward to the TBR occurring at the maximal breakpoint (see top black arrow), at which point the endemic state loses its stability and jumps back to the origin or zero state (downward pointing red arrow) and remains there as the biting rate is decreased further. These different paths followed by the parasite system for increasing versus decreasing the vector biting rate constitute the hysteresis loop effect. Figure 6B shows the equivalent loop for the system in which the vector is anopheline. Here, the loop has a considerably greater range in the space, indicating both that the emergence or re-invasion of the filarial endemic state for a similar input of infected hosts (0.1% Mf prevalence) will occur at a significantly larger vector biting rate and the subsequent state transition to the zero state will require a greater reduction of vector numbers compared to the culicine case (Fig. 6A).

## Discussion

Stability analyses of the present filarial transmission models support recent work that has shown that elimination thresholds in filarial infections depend strongly upon the nature and magnitude of density dependent processes that govern infection dynamics in the vector and host populations [5], [10]. In particular, our results underscore suggestions that the type and magnitude of these thresholds will depend on the opposing actions of positive- and negative density dependent processes acting on parasite infection dynamics. We have shown here for the first time, however, that when the major density dependent processes governing infection in the vector and host populations are of the negative density-dependent or limitation type, no worm breakpoints but only a vector biting threshold can occur in vector-borne macroparasitic diseases (Fig. 2A). In system dynamics terms, these biting thresholds would indicate a continuous transcritical transition between the extinct and positive stable parasite states, in which below the vector biting threshold a parasite-free and above which a smooth transition to a parasite endemic state occurs. Given the lack of worm breakpoints and the gradual transition to the zero- or parasite extinct state as the parasite population is depressed, it is clear that the control import of this type of threshold is that filarial eradicability will be comparatively more difficult to achieve using mass chemotherapy methods [21]. By contrast, it is only when positive density dependent processes that are likely to act on filarial infection dynamics, such as the facilitating-type larval development and adult worm mating probability functions, are added that complex system dynamics emerge, including the occurrence of worm breakpoints in addition to threshold vector biting rates, and the existence of multiple stable states separated by an unstable worm breakpoint boundary once the biting threshold is exceeded (Fig. 2B–D). An important dynamical difference of significance to filariasis elimination is that once both these biting and worm breakpoint thresholds are traversed, system transition occurs abruptly with sudden jumps to either of the zero- or endemic stable equilibrium system states. This represents a hard loss of the respective stable states [20]–[22]; however, while this phenomenon can clearly enhance parasite eradicability as a result of control, note that it can also lead to a sudden emergence of stable infection following control if the parasite can be re-introduced sufficiently to allow exceedance of these elimination thresholds.

These results have also revealed new insights regarding the relative and simultaneous effects of multiple positive density dependent processes acting at different stages of the life cycle on parasite transmission thresholds. This is most clearly appreciated in the case of the results from the stability analysis of the anopheline model, which showed that while including the adult worm mating probability function can further raise the values of both the worm breakpoint and vector biting thresholds, this combined effect only slightly changes the threshold values obtained when incorporating only the facilitating-type larval development function in the model (Fig. 2C versus D). While it is conceivable that this finding is dependent upon the specific parameters of the mating probability and larval development functions used, with the former less well supported by data compared to the latter (although our preliminary sensitivity analysis did not show great sensitivity of this result to variations in the parameters of the mating probability function used), this situation suggests that the dominant positive density dependent process acting on elimination thresholds in anopheline filariasis could be the one exerted by the facilitation-type larval development function with the adult worm mating function exerting only a weak Allee-type effect. This is further supported by the results from the culicine model which show that including only the mating function will result in much lower worm breakpoint prevalence thresholds (Fig. 2B). These findings highlight the important role that the existence of multiple Allee or positive density dependent effects acting at different population life history stages, and importantly their interactions, can play in the population dynamics and management of populations [23].

Although we have addressed only two forms of positive density dependent mechanisms here, viz. the worm mating probability and the facilitation-type larval development functions, mainly because these are the better known of such functions at present, it is relevant to note that other positive density dependent or Allee functions may also be involved in mediating filariasis transmission. One such function has been recently highlighted by the results of Duerr et al [10], who showed that including immunosuppression or infection tolerance processes could, in combination with the other positive density dependent processes, further shift parasite elimination thresholds to higher values. The involvement of immunotolerance mechanisms in lymphatic filariasis transmission has been speculated in the literature [24]–[26], but so far data that would allow parameterization of its likely effect are lacking such that we are unable to reliably consider its inclusion in this study.

The present estimates of parasite elimination thresholds, particularly in the case of the worm breakpoint prevalence value, are the first such model-based measures obtained for anopheline filariasis [5], [7]. Their values compared to those obtained for these thresholds in culicine filariasis (maximal breakpoint prevalence of 1.01% versus 0.53% and TBR of 271 versus just 9 per month), however, confirm previous more empirical-based estimates and suggestions that parasite elimination thresholds are likely to be higher in anopheline filariasis [5], [10], [27], [28], and thus that it would be easier to eliminate filariasis transmission from endemic communities, such as those found in Papua New Guinea and West Africa, exposed to anopheline vectors [5], [27]. However, as noted above, of the two positive density dependent functions shown to be directly implicated in producing this effect in this study, we were able to parameterize only the larval uptake and development functions with empirical data (Fig. 1). The functional form and parameterization of the mating probability function, by contrast, followed both the conventional formulation used previously to describe this function for the likely mating behaviour of dioecious and polygamous helminths [8], [16] and the convenient incorporation of the infection aggregation parameter as quantified from the observed relationship between community Mf prevalence and intensity (see Materials and Methods). Given that this function (despite giving rise to a weak Allee effect) is the primary cause of the occurrence of a worm breakpoint in the case of culicine filariasis, it is clear that better parameterization of this function and indeed better knowledge of worm mating behaviour in general will be required to fully address the impact of mating probabilities in filariasis transmission.

The emergence of bistable infection states above the vector threshold biting rates (TBRs) for each parasite-vector species transmission combination, in which a stable parasite-extinct state is separated from the endemic state by unstable worm breakpoint equilibria, may perhaps constitute the first important outcome of the complex dynamics that could underlie lymphatic filariasis transmission (Fig. 2). A central finding is that the unstable worm breakpoint boundaries separating the two stable states are dynamic functions of the vector biting rates above TBR for each parasite-vector infection combination. Thus, while the worm breakpoint prevalences are highest at each respective TBR, these decline markedly as vector biting rates above the TBR values increase (Fig. 2B–D). These inverse relationships have two serious implications for filariasis elimination. First, they suggest that as worm breakpoints are dependent upon the prevailing vector biting intensity, setting such thresholds for chemotherapy-based intervention programmes should take account of this factor. Second, they highlight a strategic role for vector control in filariasis elimination, as reducing vector biting rates towards the TBR values will shift the values of worm breakpoints upwards thus making achievement of elimination easier (Fig. 2B–D). In addition, since worm breakpoints are always higher at comparable vector biting rates in the case of anopheline filariasis compared to the culicine case (compare Figs. 2B and D), this role of vector control in aiding chemotherapy-based parasite elimination is likely to be more effective for the latter parasite-vector system. This is further suggested by the steeper slope of the relationship between worm breakpoints and vector biting rates obtained for culicine filariasis, whereby a small reduction in vector biting is likely to lead to a higher rise in breakpoint values compared to the more gradual rise over larger vector reductions expected in anopheline filariasis (Fig. 2B and D).

Our analyses of the sizes of the basins of attraction to either of the bistable steady states existing above respective TBRs together with assessments of transient system dynamics near equilibrium states have for the first time uncovered knowledge on another important feature related to the complex dynamics of filariasis transmission, viz the resilience of the culicine versus anopheline parasite systems to extinction-causing perturbations. First, the simulation results shown in Figure 3 indicate that, largely as a result of the lower worm breakpoint boundary, the size of the basin of attraction to the stable endemic state is larger compared to the corresponding size of the attraction basin pertaining to the extinct state in the case of the culicine-parasite system as opposed to the situation for anopheline filariasis. Given that the size of the basin of attraction around a state corresponds to the maximum perturbation that the state can undergo without a shift to an alternative stable state [20], [29], the results in Figure 3 can thus be taken to imply that culicine filariasis is more resilient to extinction-causing perturbations compared to the anopheline-parasite system. Conversely, with a comparatively larger basin of attraction to the zero-parasite state, it is clear that the anopheline-filarial system will be more robust to the re-establishment of endemic infection once elimination is achieved in an endemic area. Indeed, given the further prospect that filarial system resilience to either the endemic or extinct state could also vary dynamically with vector biting rates (Fig. 4), these findings underscore yet another strategic role for vector control in LF elimination programmes , viz. that by shrinking the size of endemic state attraction basin through reducing vector biting densities, the resilience of the endemic infection state can be reduced thereby improving the prospects of filariasis elimination.

Our analysis of the rates of change of the two filarial models near infection equilibrium points has shed further light regarding the resilience of the filarial system to perturbations. The fundamental result here is that system dynamics are shown to be slow near both the unstable breakpoint and stable endemic infection states making this parasite system resistant to state or regime shifts [30]. Such slowing of transient dynamics at unstable boundaries and before an attractor is reached implies that a complex system is able to persist in the face of relatively extreme disturbances to driving variables and parameters possibly via a greater internal ability to adapt to system perturbations [30], [31]. It also implies that although bistable filarial states may arise due to a subcritical transition (see above) when the worm breakpoint thresholds are crossed, the consequent regime change may occur much more slowly than the sudden jumps to these states generally implied by this bifurcation mechanism. The latter behaviour has clearly important implications for both the eradicability and design of elimination programmes against filariasis, viz. 1) when parasite levels are reduced, through mass chemotherapy, to levels below but *very close* to the breakpoint level, they may take a long time to fall to zero, and 2) conversely, if the parasite level is not driven below the breakpoint and remains *very close* but above it, the time taken for the system to rebound to its original endemic level may also be long. Indeed, this result suggests that the slow dynamical regions near breakpoints may themselves represent the target community parasite levels for parasite control if elimination is deemed to be unviable. Evidence for the existence of these slow dynamics in filariasis may be emerging from surveillance data in regions, such as Southern India [12], [32], where persistence of low level parasite intensity has been observed to occur despite long-term repeated mass chemotherapy of endemic communities.

The finding of the likely occurrence of significant system hysteresis in filariasis transmission dynamics in this study is unsurprising given the likely emergence of multiple parasite stable states above TBR values. The importance of this complex system phenomenon in parasite transmission dynamics and control is that endemic states may emerge at values of driving forces, such as vector biting rates, that differ from those at which the endemic states lose stability and become attracted to the extinct state (Fig. 6). The control significance of this finding is clear: if community vector biting is not reduced then following parasite reduction in humans (by chemotherapy), a small fluctuation or input of parasites into a community can cause the re-emergence of the stable filarial endemic state whereas including vector control would essentially by reducing the hysteresis loop increase the re-emergence Mf prevalence threshold and hence aid the maintenance of parasite elimination. Again, the longer and shallower hysteresis loop occurring for anopheline compared to culicine filariasis implies that vector control may be of more importance to sustaining the elimination of the former host-parasite-vector system given the wider range of vector biting values at which a small fluctuation in parasite levels can lead to the re-emergence of the endemic state (Fig. 6).

The results from analyses of the sensitivity of estimated worm breakpoint and vector biting thresholds to variations in two key infection variables that are likely to differ between communities, viz. the degree of infection aggregation and magnitude of acquired immunity, and parameter value uncertainty of an important model variable (per capita worm fecundity), clearly supports another key conclusion regarding parasite eradication gained from this study – that universally applicable values signifying parasite elimination endpoints are unlikely to exist for filariasis [5]. Rather, this result implies that the opposite – that threshold values will vary dynamically between communities depending on initial conditions of key infection parameters – is more likely to be true. An important finding, in this connection, is that significant between vector-species differences may also exist in the sensitivity of thresholds to variability in initial infection conditions and parameter uncertainty, with TBR values for anopheline-transmitted filariasis more likely to be influenced by such variability compared to culicine-vectored filariasis.

Overall, our results imply two major outcomes for the design of filariasis elimination programmes. First, complex transmission dynamics (including the eradication threshold sensitivity to initial conditions, uncertainty, existence of multiple stable states, system resilience and hysteresis) indicate that top-down command and control management practices characterized by the selection and use of single elimination targets and assumption of certainty in the knowledge of system response to perturbations (ie. essentially embodying a “one size fits all” solution) is unlikely to result in the successful elimination of parasitic infections in all regions [33], [34]. In fact, such approaches can lead to surprise and unintended or pathological consequences if pursued uncritically. This implies that more flexible and adaptive management systems that allow learning from monitoring data to revisit objectives and assumptions, resolve uncertainties in knowledge, and set more locally relevant but variable endpoint targets, will need to be developed and applied if we are to successfully accomplish the goal of parasite eradication [7], [35], [36]. This will not only require a change from the traditional normal science paradigm as embodied by command and control approaches to using a management paradigm, which embraces uncertainty, stability, resilience and dynamic variability of parasite transmission systems, but also one which allows addressing and supporting capacities for institutional learning by national disease control agencies [37], [38]. No doubt, adopting such a framework would be more exacting than the present top-down approaches but the present results indicating the existence of complex system dynamics in filariasis transmission suggest that unless this change in management is attempted, the success of the current global initiative to achieve the elimination of this disease at least would remain indeterminate. In the interim, however, we suggest that a compromise might be to use the lowest endpoint values estimated by the present models as shown in Fig. 5 and perhaps Fig. 6, to serve as targets for filariasis elimination. This may result in longer than required intervention in areas with higher endpoint thresholds leading to greater cost outlays (not to mention the ethical problems of continuing treatment of infection-free communities) but would represent a risk averse insurance against the likelihood of not accomplishing elimination in other areas with lower thresholds [39].

The second implication of our results for the design of filariasis elimination programmes highlighted throughout in this paper relates to the strategic importance of including vector control in the mass chemotherapy-based interventions being implemented currently to achieve the elimination of infection. We have shown how vector control can by (1) increasing the worm breakpoint threshold value (Fig. 2), (2) reducing the resilience of the endemic state (Fig. 4), and (3) raising the re-emergence infection threshold (Fig. 6), play a vital role not only in enhancing the prospects of achieving parasite elimination but also sustaining the parasite-free state. Together with the fact that including vector control will also reduce the number of years of intervention required to achieve parasite elimination [4], [5], our results overall thus strongly support the incorporation of vector control activities in currently running, soon to be implemented and future filariasis elimination programmes.

Although the present analyses have allowed us to dissect the complex nature of the transmission of this parasitic disease, including shedding light on how elements of complex system dynamics will impact on management strategies to affect parasite elimination, this work has also indicated several lines of future research. First, it is clear that a more complete investigation of the effects of transmission dynamics on parasite eradicability will require an examination of how stochastic effects influencing transmission processes would affect the results presented here [40]–[42]. We believe that the dynamical aspects of the current results would still hold broadly in a stochastic context but a quantification of the probabilities associated with extinction events—calculated by examining the outcomes of a large ensemble of model runs—would, however, perhaps be more realistic in guiding control programme design and management. Second, our research has also highlighted the crucial need for improved detection and determination of parameter values for all relevant components and processes occurring in parasite transmission ecology, especially those associated critically with density-dependent mechanisms governing infection transmission in different host-parasite-vector systems, if we are to more fully understand the stability, resilience and extinction dynamics of parasitic systems. In particular, we indicate here a critical need to identify and quantify more reliable mating probability functions for macroparasites, perhaps via the application of novel molecular ecological tools to parasite samples in order to reveal patterns in worm mating behaviours [43], [44]. We suggest that better management of parasite eradication founded on internal system dynamics rather than solely based on external prescription and regulation are more likely to emerge if we are able to not only successfully manage to resolve these questions but also act to increase appreciation among programme managers and policy makers of the pivotal impact of parasite transmission dynamics in underlying the eradicability of infectious diseases.

## Supporting Information

Appendix S1 (0.10 MB DOC)

## References

[1] Hotez P, Raff S, Fenwick A, Richards F, Molyneux DH (2007). Recent progress in integrated neglected tropical disease control.. Trends Parasitol.

[2] Utzinger J, de Savigny D (2006). Control of neglected tropical diseases: integrated chemotherapy and beyond.. PLoS Med.

[3] Lammie PJ, Fenwick A, Utzinger J (2006). A blueprint for success: integration of neglected tropical disease control programmes.. Trends Parasitol.

[4] Michael E, Malecela-Lazaro MN, Simonsen PE, Pedersen EM, Barker G (2004). Mathematical modelling and the control of lymphatic filariasis.. Lancet Infect Dis.

[5] Michael E, Malecela-Lazaro MN, Kabali C, Snow LC, Kazura JW (2006). Mathematical models and lymphatic filariasis control: endpoints and optimal interventions.. Trends Parasitol.

[6] Gyorkos TW (2003). Monitoring and evaluation of large scale helminth control programmes.. Acta Trop.

[7] Michael E, Malecela-Lazaro MN, Kazura JW (2007). Epidemiological modelling for monitoring and evaluation of lymphatic filariasis control.. Adv Parasitol.

[8] Michael E, Malecela-Lazaro MN, Maegga BT, Fischer P, Kazura JW (2006). Mathematical models and lymphatic filariasis control: monitoring and evaluating interventions.. Trends Parasitol.

[9] May RM (1977). Thresholds and Breakpoints in Ecosystems with a Multiplicity of Stable States.. Nature.

[10] Duerr HP, Dietz K, Eichner M (2005). Determinants of the eradicability of filarial infections: a conceptual approach.. Trends Parasitol.

[11] Chan MS, Srividya A, Norman RA, Pani SP, Ramaiah KD (1998). Epifil: a dynamic model of infection and disease in lymphatic filariasis.. Am J Trop Med Hyg.

[12] Norman RA, Chan MS, Srividya A, Pani SP, Ramaiah KD (2000). EPIFIL: the development of an age-structured model for describing the transmission dynamics and control of lymphatic filariasis.. Epidemiol Infect.

[13] Snow LC, Michael E (2002). Transmission dynamics of lymphatic filariasis: density-dependence in the uptake of Wuchereria bancrofti microfilariae by vector mosquitoes.. Med Vet Entomol.

[14] Snow LC, Bockarie MJ, Michael E (2006). Transmission dynamics of lymphatic filariasis: vector-specific density dependence in the development of Wuchereria bancrofti infective larvae in mosquitoes.. Med Vet Entomol.

[15] Bryan JH, Southgate BA (1988). Factors affecting transmission of Wuchereria bancrofti by anopheline mosquitoes. 2. Damage to ingested microfilariae by mosquito foregut armatures and development of filarial larvae in mosquitoes.. Trans R Soc Trop Med Hyg.

[16] May RM (1977). Togetherness among Schistosomes - Effects on Dynamics of Infection.. Mathematical Biosciences.

[17] Michael E, Bundy DA, Grenfell BT (1996). Re-assessing the global prevalence and distribution of lymphatic filariasis.. Parasitology.

[18] Chan MS, Guyatt HL, Bundy DA, Booth M, Fulford AJ (1995). The development of an age structured model for schistosomiasis transmission dynamics and control and its validation for Schistosoma mansoni.. Epidemiol Infect.

[19] Gunderson LH (2000). Ecological resilience - in theory and application.. Annual Review of Ecological Systems.

[20] Holling CS (1973). Resilience and stability of ecological systems.. Annual Review of Ecological Systems.

[21] Ludwig D, Walker B, Holling CS (1997). Sustainability, stability, resilience.. Conservation Ecology.

[22] Ludwig D, Jones DD, Holling CS (1978). Qualitative analysis of insect outbreak systems: Spruce-budworm and forest.. Journal of Animal Ecology.

[23] Berec L, Angulo E, Courchamp F (2006). Multiple Allee effects and population management.. Trends in Ecology and Evolution.

[24] Grenfell BT, Michael E (1992). Infection and disease in lymphatic filariasis: an epidemiological approach.. Parasitology.

[25] Maizels RM, Bundy DA, Selkirk ME, Smith DF, Anderson RM (1993). Immunological modulation and evasion by helminth parasites in human populations.. Nature.

[26] King CL (2001). Transmission intensity and human immune responses to lymphatic filariasis.. Parasite Immunol.

[27] Pichon G (2002). Limitation and facilitation in the vectors and other aspects of the dynamics of filarial transmission: the need for vector control against *Anopheles*-transmitted filariasis.. Annals of Tropical Medicine and Parasitology.

[28] Southgate BA, Bryan JH (1992). Factors affecting transmission of Wuchereria bancrofti by anopheline mosquitoes. 4. Facilitation, limitation and proportionality and their epidemiological significance.. Transactions of the Royal Society of Tropical Medicine and Hygiene.

[29] Scheffer M, Carpenter S, Foley JA, Folke C, Walker B (2001). Catastrophic shifts in ecosystems.. Nature.

[30] Walker B, Meyers JA (2004). Thresholds in ecological and social-ecological systems: a developing database.. Ecology and Society.

[31] Turner SJ, Johnson AR, Jensen ME, Bourgeron PS (2001). A theoretical framework for ecological assessment.. A Guidebook for Integrated Ecological Assessments.

[32] Stolk WA, Swaminathan S, van Oortmarssen GJ, Das PK, Habbema JD (2003). Prospects for elimination of bancroftian filariasis by mass drug treatment in Pondicherry, India: a simulation study.. J Infect Dis.

[33] Holling CS, Meffe GK (1996). Command and control and the pathology of natural resource management.. Conservation Biology.

[34] Folke C, Carpenter S, Walker B, Scheffer M, Elmqvist T (2004). Regime shifts, resilience, and biodiversity in ecostem management.. Annual Review of Ecological Systems.

[35] Holling CS (1978). Adaptive Environmental Assessment and Management.

[36] Walters CJ (1986). Adaptive Management of Renewable Resources.

[37] Funtowicz SO, Ravetz JR (1993). Science for the post-normal age.. Futures.

[38] Allison H, Hobbs R (2006). Science and Policy in Natural Resource Management.

[39] Burgman M (2005). Risks and Decisions for Conservation and Environmental Management.

[40] May R (1973). Stability and Complexicity in Model Ecosystems.

[41] Wang YH, Gutierrez AP (1980). An assessment of the use of stability analyses in population ecology.. Journal of Animal Ecology.

[42] Lande R, Engen S, Saether B-E (2003). Stochastic Population Dynamics in Ecology and Conservation.

[43] Avise JC (2004). Molecular Markers, Natural History, and Evolution.

[44] Freeland JR (2005). Molecular Ecology.

[45] Rajagopalan P (1980). Population dynamics of Culex pipiens fatigans, the filariasis vector, in Pondicherry - influence of climate and environment.. Proc Ind Nat Science Acad.

[46] Subramanian S, Manoharan A, Ramaiah KD, Das PK (1994). Rates of acquisition and loss of Wuchereria bancrofti infection in Culex quinquefasciatus.. Am J Trop Med Hyg.

[47] Hairston NG, de Meillon B (1968). On the inefficiency of transmission of Wuchereria bancrofti from mosquito to human host.. Bull World Health Organ.

[48] Ho BC, Ewert A (1967). Experimental transmission of filarial larvae in relation to feeding behaviour of the mosquito vectors.. Trans R Soc Trop Med Hyg.

[49] Evans DB, Gelband H, Vlassoff C (1993). Social and economic factors and the control of lymphatic filariasis: a review.. Acta Trop.

[50] Ottesen EA, Ramachandran CP (1995). Lymphatic Filariasis Infection and Disease - Control Strategies.. Parasitology Today.

[51] Vanamail P, Subramanian S, Das PK, Pani SP, Rajagopalan PK (1990). Estimation of Fecundic Life-Span of Wuchereria-Bancrofti from Longitudinal-Study of Human Infection in an Endemic Area of Pondicherry (South-India).. Indian Journal of Medical Research Section a-Infectious Diseases.

[52] Vanamail P, Ramaiah KD, Pani SP, Das PK, Grenfell BT (1996). Estimation of the fecund life span of Wuchereria bancrofti in an endemic area.. Transactions of the Royal Society of Tropical Medicine and Hygiene.

[53] Subramanian S, Krishnamoorthy K, Ramaiah KD, Habbema JD, Das PK (1998). The relationship between microfilarial load in the human host and uptake and development of Wuchereria bancrofti microfilariae by Culex quinquefasciatus: a study under natural conditions.. Parasitology.

[54] Subramanian S, Pani SP, Das PK, Rajagopalan PK (1989). Bancroftian filariasis in Pondicherry, south India: 2. Epidemiological evaluation of the effect of vector control.. Epidemiol Infect.

[55] Das PK, Manoharan A, Subramanian S, Ramaiah KD, Pani SP (1992). Bancroftian filariasis in Pondicherry, south India–epidemiological impact of recovery of the vector population.. Epidemiol Infect.

