# Supplementary material for: Complex Ecological Dynamics and Eradicability of the Vector Borne Macroparasitic Disease, Lymphatic Filariasis
Source: PLoS One. 2008 Aug 6;3(8):e2874. doi: 10.1371/journal.pone.0002874 (PMC2518518; doi:10.1371/journal.pone.0002874)
Supplement: Appendix S1 — (0.10 MB DOC) [file pone.0002874.s001.doc]

**Appendix S1**

The following appendix outlines the calculation performed to obtain the expression for the population-summed Mf uptake and L3 development, when the distribution of Mf is negative binomial with mean *M* and aggregation parameter *k*. The negative binomial distribution for *x* (= 0, 1, 2…) with parameter *k* and probability *p* is given by:

$P_{nb}(x;k,p)=\frac{\Gamma(k+x)}{\Gamma(k)x!}p^{k}(1-p)^{x}$ (A1)

with the moment generating function (*mgf*) of the negative binomial distribution given as:

$mgf=\left[\frac{p}{1-e^{z}(1-p)}\right]^{k}$ (A2)

Substituting$p=\frac{k}{k+M}$ into the above expression results in the use of a more easily and commonly used parameter, namely the mean (*M*) of the distribution. The mean value in the system studied here is equal to the mean community Mf load. As outlined in the main text, mosquitoes uptake Mf when they take bloodmeals from hosts and Mf become L3 larvae within these intermediate hosts. For culicine mosquitoes, the relationship between the quantity of Mf (*m*) in peripheral human blood and the number of L3 per mosquito, is given by (see text):

$L(m)=\kappa_{1}\left(1-e^{-\frac{r_{1}m}{\kappa_{S1}}}\right)$ (A3)

Replacing the expression $e^{-\frac{r_{1}}{\kappa_{S1}}}$with $e^{z}$, the quantity required for the population sum in the case of the culicine uptake function is thus given by:

$f(M)=\sum_{m=0}^{\infty} e^{zm}P_{nb}(m;k,M)$ **(A4)**

where $P_{nb}(m;k,M)$is the probability mass function of the negative binomial distribution of the variable *m* with aggregation parameter *k* and mean value *M*. This expression is identical to that required for the calculation of the moment generating function.

$mgf=\left[\frac{\frac{k}{M+k}}{1-e^{z}\left(1-\frac{k}{M+k}\right)}\right]^{k}=\frac{1}{\left[1+\frac{M}{k}\left(1-e^{z}\right)\right]^{k}}$ **(A5)**

So, for $z=\frac{r_{1}}{\kappa_{S1}}$the moment generating function, and also, therefore, the population sum, becomes:

$mgf=f(M)=\frac{1}{\left[1+\frac{M}{k}\left(1-e^{-\frac{r_{1}}{\kappa_{S1}}}\right)\right]^{k}}$ **(A6)**

Now, the uptake function associated with anopheline mosquitoes begins with a facilitation phase, and this is modelled here by squaring the *Culex* function and introducing an offset value of the Mf intensity (*m*), below which the L3 larval production is zero:

*L(m) =* 0 for *m<T*

$L(m)=\kappa_{2}\left(1-e^{-\frac{r_{2}(m-T)}{\kappa_{S2}}}\right)^{2}$ for *m*$\geq T$ **(A7)**

For the calculation of *f(M)*, the second part of the expression in brackets above is needed. Expanding this part out by squaring the bracketed expression and naming it *U(m)* gives:

*U(m)* = 1 (since *L(m)* = 0, as above)  *m<T*

*U(m)* = $2e^{\frac{r_{2}T}{\kappa_{s2}}}e^{-\frac{r_{2}m}{\kappa_{s2}}}-e^{\frac{2r_{2}T}{\kappa_{s2}}}e^{-\frac{2r_{2}m}{\kappa_{s2}}}$ , *m*$\geq T$ **(A8)**

Therefore, an extension of the *Culex* uptake argument gives:

$f(M)=\sum_{m=0}^{\infty} U(m)P_{nb}(m;k,M)=\sum_{m=0}^{T} U(m)P_{nb}(m;k,M)+\sum_{m=T}^{\infty} U(m)P_{nb}(m;k,M)$ **(A9)**

Using the moment generating function outlined earlier, the above expression becomes:

$$f(M)=\sum_{m=0}^{T} P_{nb}(m;k,M)+\left[\frac{2e^{\frac{r_{2}T}{\kappa_{s2}}}}{\left[1+\frac{M}{k}\left(1-e^{-\frac{r_{2}}{\kappa_{s2}}}\right)\right]^{k}}-\frac{e^{\frac{2r_{2}T}{\kappa_{s2}}}}{\left[1+\frac{M}{k}\left(1-e^{-\frac{2r_{2}}{\kappa_{s2}}}\right)\right]^{k}}\right]\sum_{m=T}^{\infty} P_{nb}(m;k,M)$$

**(A10)**

When the above quantity is calculated, it is important to note that the negative binomial distribution is an integer-valued probability mass function, but that the offset parameter may not be. A simple continuous approximation is therefore made, in which the probability mass is assumed to be uniformly distributed between integers and integer values are summed up to the decimal fraction, whereupon the remaining proportion of the probability is calculated as a fraction of the mass lying between the subsequent two integers.
